# Long-Term Environmental Methylmercury Exposure Is Associated with Peripheral Neuropathy and Cognitive Impairment among an Amazon Indigenous Population

**DOI:** 10.3390/toxics12030212

**Published:** 2024-03-12

**Authors:** Bruno H. Rebouças, Gabriel T. Kubota, Rogério A. A. Oliveira, Bruna D. Pinto, Roberta M. Cardoso, Ana C. S. Vasconcellos, Paulo C. Basta

**Affiliations:** 1 Department of Neurology, Hospital das Clínicas, Faculty of Medicine, University of São Paulo (USP), Sao Paulo 05403-000, Brazil; 2 Pain Treatment Center, São Paulo State Cancer Institute, Sao Paulo 01246-000, Brazil; 3 Laboratory of Professional Education in Health Surveillance, Polytechnic School of Health Joaquim Venacio (EPSJV), Oswald Cruz Foundation (Fiocruz), Rio de Janeiro 21040-900, Brazil; 4 Program of Post-Graduation in Public Health and Environment, National School of Public Health (ENSP), Oswald Cruz Foundation (Fiocruz), Rio de Janeiro 21040-900, Brazil; paulobasta@gmail.com; 5 Department of Endemic Diseases Samuel Pessoa, Escola Nacional de Saúde Pública Sergio Arouca, Oswald Cruz Foundation (Fiocruz), Rio de Janeiro 21041-210, Brazil

**Keywords:** mercury poisoning, neurotoxicity syndromes, indigenous peoples, cognitive dysfunction, methylmercury compounds, polyneuropathy, environmental health

## Abstract

Widespread contamination of the Amazon basin with mercury has been reported to occur since at least the mid-80s due to heavy gold mining activity. Although initial studies have indicated that this may lead to deleterious neurological consequences to the indigenous populations living in the region, further research is needed to better characterize the neurological burden of such long-term exposure. With this aim, a cross-sectional exploratory study has been conducted with the Yanomami indigenous population residing in a northern Amazon region. All participants underwent a structured interview; detailed neurological examination, including assessment for cognitive, motor, coordination, and sensory functions; and laboratorial testing for serum hemoglobin, blood glucose, and methylmercury levels in hair samples. This study enrolled 154 individuals of 30.9 ± 16.8 years of age, of which 56.1% were female. Mean methylmercury levels in hair were 3.9 ± 1.7 µg/g. Methylmercury levels in hair > 6.0 µg/g were found in 10.3%. Among participants with hair methylmercury levels ≥ 6.0 μg/g, the prevalences of peripheral neuropathy and reduced cognitive performance were, respectively, 78.8% (95%CI 15–177%, *p* = 0.010) and 95.9% (95%CI 16–230.8%, *p* = 0.012) higher than those of individuals with lower levels. These results suggest that chronic mercury exposure may lead to significant and potentially irreversible neurotoxicity to Yanomami population living in the northern Amazon basin.

## 1. Introduction

Neurotoxicity due to methylmercury (MeHg), an organo–mercurial compound, was classically described in Minamata and Niigata disasters, more than 50 years ago [1]. A massive poisoning of these Japanese communities took place due to human ingestion of fish contaminated with high levels of MeHg. A wide range of serious, life-threatening, and incapacitating neurological symptoms and signs were described and named as Minamata disease. Paresthesia and other somatosensory deficits, ataxia, movement disorders such as dystonia and parkinsonism, dysarthria, visual field alterations, and hearing problems happened in a scenario that lasted for decades, and affected several generations [2,3].

Following this episode, in the early 1970s, an acute exposure to high levels of MeHg in Iraq led to the intoxication of thousands of adults and children, and hundreds of deaths in a short period of time [4,5]. By then, improvements of the atomic absorption spectrometry technique for analyzing mercury in human tissue (e.g., hair and blood) allowed for more precise quantification of exposure to this substance. The range of MeHg levels necessary to bring about signs of toxicity could be better understood [6], and a World Health Organization (WHO) consensus established that the neurological effects related to MeHg poisoning could be found in 5% of adult individuals who ingest 3 to 7 µg/kg/day, which corresponds to levels of 200 µg/L in blood and 50 µg/g in hair samples [1].

Meanwhile, in the Amazon region of South America, decades of non-regulated and undeterred gold mining activity have launched significant quantities of metallic mercury into the rivers, leading to the progressive bioaccumulation of this substance in the food chain [7]. Part of the metallic mercury dumped into rivers is converted into mercuric ions when it encounters other ions dissolved in the water. The mercury ionic forms adhere to particles suspended in water that tend to move to the bottom of rivers and accumulate in sediments. In the sediment, methanogenic bacteria living in anoxic areas absorb these ions and transform them into MeHg. Due to its lipophilic characteristics, MeHg is quickly absorbed by the microscopic organisms that make up the aquatic biota (e.g., phytoplankton, zooplankton). Such microscopic organisms serve as food for larger ones (e.g., fish larvae), which in turn are consumed by small fishes. In this process, methylmercury is bioaccumulated in aquatic life forms and transferred throughout trophic levels (i.e., biomagnified) until it reaches organisms at the top of the trophic chain (for example, carnivorous fish) [8,9].

In addition to gold mining, the construction of dams, deforestation, and fires have also altered the local mercury cycle, contributing to this process [10,11,12,13,14]. Notably, since the mid-90s, samples from various fish species have been demonstrated to contain Hg concentrations above 1.0 mg/kg [15]. Once fish is the main protein source of many indigenous populations in the Amazon, they became contaminated with this substance. However, differently from the abovementioned episodes in Japan and Iraq, this exposure occurred at much lower levels and for a longer period of time [16,17,18,19]. Despite the increasing expansion of gold mining in the Amazonian indigenous lands (IL), few studies have aimed at investigating the exposure of indigenous people to Hg and its health impacts. Covering this topic in the Yanomami indigenous land, there are only three previous studies [9,20,21]. All these studies revealed that the average Hg levels, assessed by biological exposure markers, were above the limits recommended by several international health agencies (e.g., FAO/WHO, U.S.EPA, NRC, Health Canada), as a result of the artisanal gold mining as the main exposure source [22,23,24]. Furthermore, a recent study published by Vasconcellos et al. (2022) reinforces these findings, once it pointed to high Hg concentrations in fish collected from rivers that cross the Yanomami Indigenous Land, in the state of Roraima, Brazil [25]. Fish such as Filhote/Piraíba, Piracatinga, Pirandirá, and Barba-chata, which are much appreciated by residents of this region, had total Hg levels above 1.0 μg/g and therefore pose a high health risk [25].

Moreover, initial research carried out in this region indicated that, even at lower levels, MeHg may prove to have significant neurotoxic consequences to these indigenous populations. Psycho–behavioral changes, cognitive deterioration, somatosensory, and color vision abnormalities have been described and associated with MeHg levels measured in the exposed population [26]. These symptoms fall in line with the classic descriptions of massive MeHg intoxication, as seen in Minamata disease [27]. However, these initial studies are limited by relatively small samples, methodological issues, and most were concentrated in the Tapajos river area, a small region within the south part of the Amazon basin [15].

The Yanomami IL located in the northern Brazilian Amazon has harbored significant gold-mining activities in the past decades, and therefore has possibly been subjected to significant MeHg environmental poisoning. However, the severity and neurological consequences of the long-term exposure to this substance for the local indigenous population are yet to be investigated. Therefore, an exploratory observational study was undertaken, aimed at characterizing the extension of MeHg exposure and its possible neurological burden to these traditional communities.

## 2. Materials and Methods

### 2.1. Study Design

This was a cross-sectional study aimed at evaluating the neurological consequences of long-term MeHg exposure among the Yanomami indigenous population of the Brazilian Amazon. It is part of a larger multidisciplinary and inter-institutional effort, led by the Oswaldo Cruz Foundation, aimed at assessing the characteristics, severity, and consequences of MeHg contamination due to local gold mining activity in the Brazilian IL of the Amazon rainforest [7,27]. Data collection took place in the Northwest region of Roraima state, Brazil, during October 2022. The research protocol was reviewed and approved by the Institutional Review Board (#6.148.688), and all subjects provided written informed consent before enrolment.

### 2.2. Sample Population and Research Site

A census was performed among the indigenous Yanomami population of the upper Mucajaí river, in the Northwest region of the Brazilian Amazon. All individuals aged ≥ 12 years old were invited to participate; therefore, probabilistic sampling methods were not employed. This population amounted to a total of 358 registered subjects, living within nine separate villages across this region. The data collection site for this research was centered in Lasasi, one of these villages. All interviews and instructions were conducted in Brazilian Portuguese, and translation to the Yanomami language was offered by a local guide or health agents when necessary.

### 2.3. Clinical Evaluation and General Physical Examination

All participants underwent a structured interview that obtained data about general socio-demographic features and medical history. Following that, their weight and height were measured with an electronic scale and a vertical anthropometer, respectively [27]. Blood pressure was assessed with an automatic pulse blood pressure monitor Omron Model Hem-631INT (Omron Healthcare INC, Lake Forest, IL, USA), with the subject in the sitting position with both feet flat on the floor and the left forearm resting on the anterior part of the chest. Blood pressure was measured twice, and the mean systolic (SBP) and diastolic (DBP) were recorded [27].

### 2.4. Neurological Examinations

A neurological examination was performed according to a standardized protocol, by three previously trained neurologists (BDP, BHR, and RAAO), working as a team. Disagreements were resolved by discussion until consensus was reached. The examination protocol included testing for cognitive, motor, coordination, and sensory functions.

#### 2.4.1. Cognitive Evaluation

The Brief Cognitive Screening Battery (BCSB) [28] and semantic verbal fluency test (S-VFT) in the animal category [29] were used for overall cognitive assessment. Both these instruments have been previously validated for the Brazilian population [30,31], and were considered appropriate for assessing populations with a low level of formal education and the time restraints of this research.

The BCSB is an instrument developed by the Department of Neurology of the University of Sao Paulo, Brazil [30]. It consists of naming and learning 10 simple images of well-known objects, presented in a single picture, recalling them after a 5-min interval, and then identifying them among a set of other images. As per the recommended test protocol, during the 5-min interval the VFT was applied [30]. This instrument evaluates the following cognitive functions: (i) visual perception and naming, (ii) short-term episodic memory and learning; (iii) operational memory and executive functions. An abnormal result was considered as the inability to recall ≥ 6 images after the 5-min interval [30].

The S-VFT in the Animal category requires the participant to name as many animals as possible within 1 min. This screening cognitive test assesses mainly language, semantic memory, and executive functions. The individuals received the following instructions: “*you must say the animal names you remember, as quickly as possible. Any animal will do, four-legged, fish, birds, the more you say, the better*”. According to previously published work with the Brazilian population, the cutoff for S-VFT was ≥9 [31,32].

#### 2.4.2. Motor Function and Coordination

Muscular strength was evaluated in the proximal and distal segments of all four limbs, and was classified according to the Medical Research Council Scale [33]. A score ≤ 4 in any segment was considered as abnormal. Muscle rigidity was examined through passive mobilization of the limbs; and bradykinesia (slowed movement) through finger tapping maneuvers, facial expression assessment, and general spontaneous mobilization. Coordination was evaluated with finger-to-nose, heel-to-knee, and diadochokinesis (ability to make alternating quick movements) tests. The deep osteotendinous ankle reflex was tested with the Babinski hammer bilaterally. Toe amyotrophy was investigated through inspection.

#### 2.4.3. Balance and Gait Evaluation

For static balance assessment, subjects were instructed to stand with their feet as close as possible and remain still for 1 minute. Then, they were told to close their eyes while remaining standing (i.e., Romberg test). In case significant oscillations or falls occurred, or the subject needed support to remain standing (e.g., cane), balance was considered abnormal. Following this, the subjects were instructed to walk in a straight line for 6 m, turn around, and walk back the same distance, and their gait was inspected.

#### 2.4.4. Sensory Testing

Our somatosensory system has three basic types of sensory receptors that detect different types of external stimuli [34]. These include mechanoreceptors that detect light touch, vibration, pressure, and texture; nociceptors that detect pain; and thermoreceptors that detect temperature [34]. To evaluate these somatosensory modalities, the following instruments were used:

1. Sharp nickel-plated pin (Bacchi^®^ number 29): metal pin, commonly used in sewing, but which can be used for physical neurological examination and assessment of pin-prick pain sensitivity.
2. Von Frey 10 g monofilament: also known as an esthesiometer, its function is to measure the tactile sensitivity of the skin. It is a nylon monofilament that bends when it is applied with certain force to the skin, so that it always applies the same force to the skin regardless of the examiner.
3. Dry cotton wad: to determinate the dynamic tactile sensitivity (i.e., sensitivity to brushing touch).
4. 128 Hz tuning fork: An instrument used for tuning musical instruments due to its ability to vibrate at a specific frequency. In neurological examination, this instrument can be used at low frequencies to evaluate vibratory sensitivity. Furthermore, as it is a metallic object and has a cold temperature, it can also be employed to assess thermal sensitivity to cold during physical examinations.

The sensitivity in all these modalities was tested with the instruments above in a comparative manner between the upper and lower limbs, comparing one side with the other, as well as comparing proximal and distal levels. The sharp nickel-plated pin, von Frey monofilament, dry cotton wad, and 128 Hz tuning fork were used on intact healthy skin of the feet, thighs, hands, and shoulders, assessing pain, tactile, and thermal sensitivity, respectively. Additionally, deep vibratory sensitivity was evaluated using the vibrating 128 Hz tuning fork, applied to the bony prominences of the phalanges of the fingers and toes. The sensitivity was classified as abnormal if altered in at least one parameter, when compared with the contralateral side or the other assessed region of the body segment (i.e., distal or proximal). These techniques for sensory evaluation have been previously described and are widely used for bedside neurological examination [35,36].

#### 2.4.5. Cranial Nerve Examination

The following parameters were evaluated: visual campimetry by confrontation, pupil reflex and external ocular motricity, facial motricity and symmetry, facial sensibility, palate elevation, gag reflex, and tongue motricity.

#### 2.4.6. Peripheral Neuropathy Diagnosis

Peripheral neuropathy was diagnosed according to the current criteria from the American Academy of Neurology, for epidemiological research [37]. Therefore, this diagnosis was established in case of: report of neuropathic symptoms in the distal regions of the limbs; in addition to decreased or absent ankle reflex, decreased distal sensation at examination, or distal muscle weakness or atrophy [37].

### 2.5. Blood Testing

Serum hemoglobin was measured with the HemoCue^®^ device (HemoCue^®^, model HB 301-System, Angelholm, Sweden) without the need to collect and store venous blood samples. Hemoglobin levels were considered abnormal if ≤14 g/dL for men; ≤12 g/dL for women in general; and ≤11 g/dL for pregnant women [38]. To collect whole blood, the fourth digit was punctured with a lancet until a single drop of blood was obtained (approximately 50 microliters). This blood sample was delicately transferred through capillarity to a glass microcuvette specific for the HemoCue^®^ device. When the microcuvette was filled with blood, it was inserted into the equipment for reading. This device is factory-calibrated according to the reference method of the International Council for Standardization in Hematology [39].

Blood glucose levels were measured with the Accu-Chek Active^®^ blood glucose monitor (Roche, Indianapolis, IN, USA) during the clinical assessments, without established time interval since the last meal or fasting requirement. This device was used according to the manufacturer’s specifications and had a measurement range of 10 to 600 mg/dL. The glucose measurements were performed with whole blood, collected from the tip of the fourth digit with a specific needle, and subsequently transferred to the analysis device.

### 2.6. MeHg Exposure Assessment

According to the guidance published by the World Health Organization [40], and described by other authors [41,42], hair samples are considered the best biomarker of MeHg human exposure, because almost all Hg detected in this biological matrix is in the MeHg form. In addition, because the main MeHg exposure route observed in the studied population is the consumption of MeHg-contaminated fish, and almost all Hg present in the fish muscle is in the MeHg form, hair samples were used as its exposure biomarker [43,44,45]. Therefore, we assumed that all Hg present in hair samples was MeHg.

The hair samples were collected from the occipital region, with the aid of stainless- steel dissection scissors. The samples were stored in individually identified paper envelopes and sent for total mercury concentration (THg) analysis in the Toxicology Laboratory, Environmental Section of the Instituto Evandro Chagas (IEC), in Ananindeua municipality, in Pará State, Brazil.

In the laboratory, before starting the analyses, hair samples were repeatedly washed with a neutral detergent (diluted 100-fold) (Extran detergent, Merck KGaA, Darmstadt, Germany) to remove any exogenous contamination. After drying, the samples were transferred into a 20 mL vial and cut into an approximately powdery state with dissection scissors to make a homogenized sample before weighing (10–20 mg of hair are necessary for this analysis). This methodology comprises chemical opening, wet digestion, and subsequent reduction with a 10% SnCl_2_ solution to quantify total Hg in a cold vapor atomic absorption spectrometry with a photo-absorption cell for the measurement of absorbance at 253.7 nm (CVAAS) (Mercury Analyzer, Model Hg-201 Semi-automated Mercury Analyzer, Sanso Seisakusho Co., Ltd., Tokyo, Japan). All stages of this analysis method are presented in detail in the “Mercury Manual Analysis” published by the Japanese Ministry of the Environment in 2004 (http://nimd.env.go.jp/english/research/result/analysis_manual/; accessed on 25 January 2024).

The protocols for Quality Assurance (QA)/Quality Control (QC) included the follow- ing parameters: (i) a method blank; (ii) a 6-point calibration curve (concentration ranging from 0.4 to 4 ng/g); (iii) the Human Hair Certified Reference Material (IAEA-86), whose average recovery rate was 101% (n = 8, recovery ranging from 83.4 to 106.6%) from the International Atomic Energy Agency; and (iv) the relative standard deviation (RSD) of 8.32%. Sample replicates (n = 10), whose RSD was 2.49%, were also randomly selected. The detection and quantification limits (LOD/LOQ) obtained were 0.0083 ng/mg and 0.027 ng/mg, respectively.

### 2.7. Statistical Analyses

Firstly, general demographic features, physical and neurological examination findings, and laboratory results were compared between individuals according to MeHg exposure levels found in their hair samples. For this analysis, the hair MeHg concentrations of 2.0 µg/g and 6.0 µg/g were considered thresholds for neurological outcomes, because previous studies already indicated these limits as relevant for other Hg exposed populations [46,47]. Following this, subjects were grouped according to the presence of peripheral neuropathy and a reduced performance at cognitive testing. The latter was defined as a score < 6 at the delayed recall phase of the BCSB or a score < 9 at the S-VFT. Likewise, demographical, physical, and neurological examination and laboratorial data were compared between these groups.

For these analyses, categorical variables were presented as total count and percentages, and compared with chi-square test, or Fisher’s exact test when appropriate. Meanwhile, continuous variables were summarized as mean ± standard deviation (minimum–maximum), and their distributions were classified as parametric or not through the Kolmogorov–Smirnov and Shapiro–Wilk tests, and visual inspection of histograms and Q–Q plots. Parametric variables were compared with the Student’s *t*-test, and non-parametric ones with the Mann–Whitney U test. On the other hand, for analyzing the correlation between continuous variables, Pearson’s or Spearman’s correlation tests were used, when appropriate. Statistical significance was set at *p* < 0.05. Also, to evaluate the accuracy of hair-derived MeHg levels in identifying peripheral neuropathy and reduced cognitive performance in this indigenous population, a Receiver Operating Curve (ROC) was used, and the area under the curve with its 95% confidence interval (95%CI) were estimated. The ROC curve is a graphical plot that illustrates the performance of a test or variable in predicting a given outcome, at varying threshold values. The larger the area under the ROC curve, the higher the prediction accuracy.

Furthermore, to better characterize the association between the evaluated variables and the addressed clinically relevant neurological outcomes (i.e., peripheral neuropathy and a reduced performance at cognitive testing), a Poisson regression model was used. Prevalence Ratio (PR), with its respective 95%CI, was used as the association measure. For such, firstly univariate analyses were conducted for each independent variable. Variables found to be significantly associated with the studied neurological outcomes were then included in multivariate model analyses. For these latter, multicollinearity was assessed by calculating the tolerance values. Tolerance < 0.1 was considered as indication of multicollinearity [48].

All statistical analyses were performed using the software Statistical Package for the Social Sciences version 20.0.0 (SPSS Inc., Chicago, IL, USA).

## 3. Results

### 3.1. Studied Population

Of the total number of registered individuals older than 12 years old living in the Mucajaí river basin, 154 (43.0%) volunteered to participate, including almost all those who resided at the village in which the data collection center was located. Female sex accounted for 87 (56.1%) individuals, mean age was 30.9 ± 16.8 years old, and only 11 (7.1%) subjects were ≥65 years old. Reported previous medical conditions were reported by only 17 (11%). An abnormal blood pressure was present in 3 (1.9%) and mean BMI was 22.5 ± 3.1 kg/m^2^. Mean blood glucose levels was 94.2 ± 17.7 mg/dL (ranging from 56 to 150 mg/dL), and mean Hb levels was 13.6 ± 1.4 g/dL (ranging from 10.4 to 17.2 g/dL).

Altered scores for the S-VFT and the delayed recall phase of the BCSB were found in 53 (34.2%) and 9 (5.8%) individuals, respectively. Furthermore, abnormal findings in the remaining of the neurological examination were detected in 53 (34.2%). The most common findings were: abnormal nociception (n = 25, 16.1%) and thermal sensitivity (n = 23, 14.8%) in the lower limbs, altered ankle reflex (n = 26, 16.8%), and an abnormal gait (n = 12, 7.7%).

### 3.2. Exposure to Methylmercury

MeHg measurements from hair samples were available for all but one of the studied individuals. Presence of MeHg was observed in all subjects, and mean MeHg levels were 3.9 ± 1.7 μg/g. There was no correlation between these levels and age (r_s_ = 0.137, *p* = 0.091). Hair MeHg levels > 6.0 μg/g were observed in 16 (10.3%), and were significantly associated with abnormal lower limb nociception (37.5% vs. 13.9%, *p* = 0.027) and peripheral neuropathy (56.2% vs. 42.1%, *p* = 0.041; Table 1). A statistical trend for association with reduced cognitive performance was also observed (66.7% vs. 35.9%, *p* = 0.059). On the other hand, MeHg levels > 2.0 μg/g were found in the majority of individuals (n = 134, 86.5%), and were associated with older age (32.2 ± 17.0 vs. 22.6 ± 12.6, *p* = 0.008; Table S1).

### 3.3. Peripheral Neuropathy

Peripheral neuropathy was detected in 47 (30.3%) individuals. This diagnosis was associated with older age (39.7 ± 20.1 vs. 27.1 ± 13.6 years old, *p* < 0.001) and Hg levels > 6.0 μg/g (19.1% vs. 6.6%, *p* = 0.041; Table 2). In the adjusted Poisson regression model, each additional year of age was associated with a 2.6% (95%CI 1.4–3.7%) increase in the prevalence of this condition; and a MeHg > 6.0 μg/g with an increase of 78.7% (95%CI 15–177%) in this frequency (Table 4). No evidence of multicollinearity between these two variables was found.

However, MeHg exposure levels did not have statistically significant discriminatory capability for identifying peripheral neuropathy (area under the ROC curve = 0.571, 95%CI 0.464–0.678).

### 3.4. Reduced Cognitive Performance

A reduced cognitive performance was observed in 54 (34.8%) of subjects. This was associated with lower SBP (104.7 ± 9.9 vs. 110.3 ± 11.3 mmHg, *p* = 0.004), lower DBP (67.2 ± 8.6 vs. 72.2 ± 9.8 mmHg, *p* = 0.003), lower Hb levels (13.3 ± 1.3 vs. 13.8 ± 1.4 g/dL, *p* = 0.034), and higher MeHg levels (4.34 ± 1.65 vs. 3.54 ± 1.53μg/g, *p* = 0.002; Table 3). A statistical trend was also observed between reduced cognitive performance and an MeHg > 6.0 μg/g (14.8% vs. 4.7%, *p* = 0.059). In the adjusted Poisson regression model, each additional 1.0 g/dL in Hb levels were associated with a 16.5% (95%CI 2.9–28.1%) reduction in the prevalence of abnormal cognitive performance; and an Hg > 6.0 μg/g with an increase of 95.9% (95%CI 16–230.8%) in this frequency (Table 4). No evidence of multicollinearity between these two variables was found.

**Table 3:** Demographic characteristics and laboratory findings between subjects with and without reduced cognitive performance in the Yanomami indigenous population, 2022.

	Reduced Cognitive Performance (n = 54)	Normal Cognitive Performance (n = 86)	*p*
Female gender ^A^	35 (64.8%)	45 (52.3%)	0.146
Age (years)	30.7 ± 17.2 (12–75.7)	30.9 ± 16.6 (12–74.7)	0.891
Monthly income (R$)	1415.69 ± 931.00 (400–3450)	1273.32 ± 970.03 (0–3000)	0.330
BMI (kg/m^2^)	22.3 ± 2.6 (17.2–28.8)	22.8 ± 3.2 (17.1–33.1)	0.325
SBP (mmHg)	104.7 ± 9.9 (83–130)	110.3 ± 11.3 (86.5–145.5)	0.004 *
DBP (mmHg)	67.2 ± 8.6 (51–92.5)	72.2 ± 9.8 (41.5–96)	0.003 *
Abnormal blood pressure ^A,B^	1 (1.9%)	1 (1.2%)	1.000
Hb (mg/dL)	13.3 ± 1.3 (10.4–16.4)	13.8 ± 1.4 (10.6–17.2)	0.034 *
Serum glucose levels (mg/dL)	94.2 ± 15.4 (61–136)	92.9 ± 18.1 (56–150)	0.542
Serum glucosis > 126 mg/dL ^A^	7 (13.0%)	11 (12.8%)	0.976
Peripheral neuropathy ^A^	21 (38.9%)	23 (26.7%)	0.132
Hair MeHg levels (μg/g)	4.34 ± 1.65 (1.15–7.50)	3.54 ± 1.53 (1.17–10.11)	0.002 *
Hair MeHg > 2 μg/g	73 (84.9%)	49 (90.7%)	0.314
Hair MeHg > 6 μg/g	8 (14.8%)	4 (4.7%)	0.059

Values presented as mean ± standard deviation (minimum–maximum), unless otherwise specified. ^A^ Values presented as n (%). ^B^ An abnormal blood pressure was considered to be a systolic arterial pressure ≥ 140 mmHg and/or a diastolic blood pressure ≥ 90 mmHg. A reduced cognitive performance was considered to be a score < 6 at the delayed recall phase of the Brief Cognitive Screening Battery or a score < 9 at the verbal fluency test. * *p* < 0.05. MeHg—methylmercury; BMI—body mass index; SBP—systolic blood pressure; DBP—diastolic blood pressure; Hb—hemoglobin levels; Hg—mercury.

**Table 4:** Poisson regression for predicting the presence of peripheral neuropathy and reduced performance at cognitive testing in the Yanomami indigenous population, 2022.

Peripheral Neuropathy						
	Prevalence Ratio (Crude)	95%CI	*p*	Prevalence Ratio (Adjusted)	95%CI	*p*
Female gender	1.040	0.642–1.684	0.874	---	---	---
Age (years)	1.027	1.016–1.038	<0.001 *	1.026	1.014–1.037	<0.001 *
BMI (kg/m^2^)	1.040	0.967–1.120	0.9290	---	---	---
Abnormal blood pressure ^A^	2.207	0.956–5.096	0.064	---	---	---
Hb (g/dL)	1.024	0.857–1.224	0.792	---	---	---
Serum glucose levels (mg/dL)	0.999	0.986–1.011	0.999	---	---	---
Serum glucose > 126 mg/dL	0.714	0.318–1.606	0.416	---	---	---
Hair MeHg levels (μg/g)	1.143	1.006–1.299	0.040 *	---	---	---
Hair MeHg > 2 μg/g	0.810	0.426–1.542	0.522	---	---	---
Hiar MeHg > 3.7 μg/g	1.304	0.807–2.109	0.259	---	---	---
Hair MeHg > 6 μg/g	2.028	1.218–3.376	0.007 *	1.787	1.150–2.777	0.010 *
**Reduced Cognitive Performance**						
	**Prevalence Ratio (Crude)**	**95%CI**	***p***	**Prevalence Ratio (Adjusted)**	**95%CI**	***p***
Female gender	1.382	0.883–2.160	0.156	---	---	---
Age (years)	1.000	0.987–1.012	0.962	---	---	---
BMI (kg/m^2^)	0.960	0.909–1.014	0.142	---	---	---
Abnormal blood pressure ^A^	1.292	0.318–5.251	0.720	---	---	---
Hb (g/dL)	0.846	0.729–0.982	0.028 *	0.835	0.719–0.971	0.019 *
Serum glucose levels (mg/dL)	1.003	0.991–1.015	0.662	---	---	---
Serum glucose > 126 mg/dL	1.009	0.543–1.878	0.976	---	---	---
MeHg levels (μg/g)	1.186	1.048–1.342	0.007 *	---	---	---
Hair MeHg > 2 μg/g	1.446	0.666–3.141	0.352	---	---	---
Hair MeHg > 3.7 μg/g	1.664	1.077–2.571	0.022 *	---	---	---
Hair MeHg > 6 μg/g	1.855	1.169–2.945	0.009 *	1.959	1.160–3.308	0.012 *

A reduced cognitive performance was considered to be a score < 6 at the delayed recall phase of the Brief Cognitive Screening Battery or a score < 9 at the verbal fluency test. ^A^ An abnormal blood pressure was considered to be a systolic arterial pressure ≥ 140 mmHg and/or a diastolic blood pressure ≥ 90 mmHg. * *p* < 0.05. 95%CI—95% confidence interval; BMI—body mass index; Hb—hemoglobin levels; MeHg—methylmercury.

**Figure 1:**
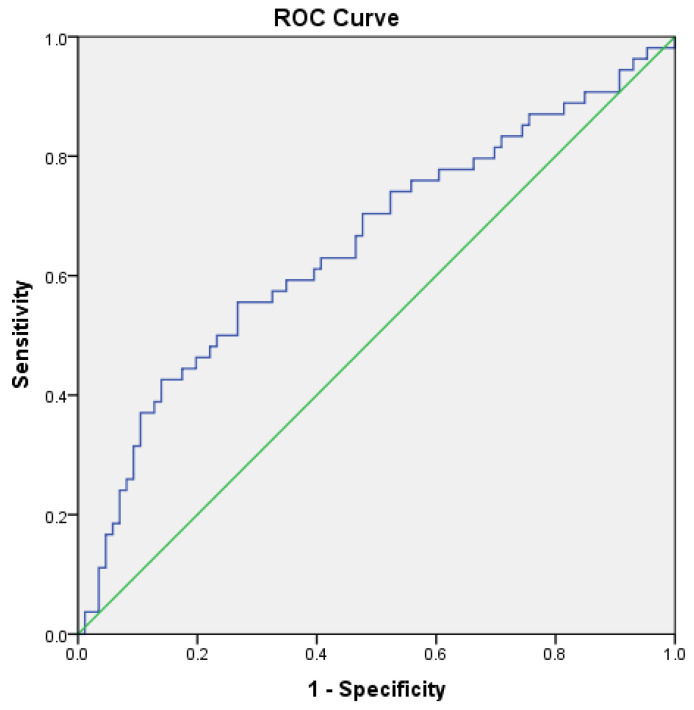
ROC curve for predicting reduced cognitive performance according to hair MeHg levels of individuals from the Yanomami indigenous population, 2022. The blue line is the Receiver Operating Characteristic curve, and the green one is the diagonal reference line.

MeHg exposure levels allowed for the prediction of reduced cognitive performance with an area under the ROC curve of 0.652 (95%CI 0.556–0.749; Figure 1). For this purpose, a MeHg level > 6.0 μg/g was found to have a sensitivity (Se) of 13.3% and a specificity (Sp) of 95.3%; while a MeHg level > 2.0 μg/g presented with an Se of 88.9% and Sp of 15.1%. The optimum MeHg detection threshold was estimated to be 3.7 μg/g, which demonstrated an Se of 55.3% and Sp of 54.7%.

## 4. Discussion

This exploratory cross-sectional study was the first to have assessed the neurotoxic effects of long-term MeHg exposure among individuals living in the Yanomami IL, at the Mucajaí river basin. Exposure to MeHg was found in all studied subjects, with mean hair levels of 3.9 ± 1.7 μg/g. In the group with MeHg levels of >6.0 μg/g, the prevalence of peripheral neuropathy and reduced cognitive performance were, respectively, 78.7% (95%CI 15–177%) and 95.9% (95%CI 16–230.8%) higher than among those with lower levels. Notably, our sample was composed of mostly young people, with few comorbidities that may otherwise lead to these neurological conditions.

Neurotoxicity attributed to MeHg is mostly due to its biomagnification capacity in aquatic trophic chains and its ability to overcome blood–brain and placenta barriers. Human exposure to MeHg commonly occurs through the consumption of contaminated fish and seafood [27]. Notably, fish is a cornerstone diet of many indigenous populations in the Amazon, and evidence accumulated since the mid-90s have demonstrated elevated MeHg concentrations in diverse fish species of this region, well above the safety limits established by the World Health Organization (WHO) [15,27,49]. MeHg present in contaminated food is readily absorbed by the digestive system and is distributed to virtually all body tissues [27,50]. Upon reaching the central nervous system, MeHg can undergo a demethylation process and mercuric ions (Hg^+2^) accumulate in the nervous tissue, causing irreversible damage [27,50]. Experimental evidence suggests that this may be the result of mitochondrial dysfunction, abnormal release of excitatory amino acids, and alterations of proteomic expression, which eventually results in increased oxidative stress, cellular dysfunction, and death [27,50,51].

However, determining what long-term MeHg exposure levels constitute a significant risk for neurological and other health-related conditions remains a challenge. In fact, reference levels vary between international health agencies, and do not necessarily reflect biological safety parameters. For example, the Food and Agriculture Organization (FAO/WHO) recommends maximum intake doses of 3.3 μg/kg/week for the general population, which corresponds to hair MeHg levels of 4.5 μg/g [52]. Yet, although our study has found a significant association between MeHg > 6.0 μg/g and both cognitive impairment and higher prevalence of peripheral neuropathy, these levels only had low-to-moderate accuracy for detecting the former, and no significant accuracy at all for the latter. This may be explained by variations of individual susceptibility, possibly influenced by several factors. Interestingly, polymorphisms of genes belonging to the metallothionein, selenoprotein, and xenobiotic transporter protein superfamilies have been associated with MeHg levels in human hair samples [53]. Characteristics of exposure besides dosage, such as length, age, and pattern (continuous versus intermittent) may also play relevant roles in individual susceptibility [32]. Alternatively, it is possible that other factors, unaddressed by this study, may have had a more significant role in producing cognitive impairment and peripheral neuropathy than MeHg exposure in the studied population.

Understanding of the neurological consequences of exposure to MeHg comes from episodes of large-scale environmental poisonings that occurred in the Minamata Bay and Yatsushiro Sea, Japan, between 1950 and 1968 [54], and in the rural region of Iraq during the winter of 1971–1972 [4]. In severe cases, a neurological disorder known as Hunter–Russell syndrome occurs, including visual field constriction, hearing impairment, ataxia, and sensory disorders. In milder cases, however, only somatosensory neuropathy may be observed, as well as psychomotor deficits [55].

On the other hand, data about the neurological consequences of long-term exposure to lower concentrations of MeHg are still lacking. In fact, available studies within this context are frequently hampered by small samples, absence of control populations, inadequate MeHg measurement methods, and other methodological issues [15,56]. Nonetheless, they suggest possible negative impacts in a variety of neurological functions, stemming from cognitive, motor, and coordination, to hearing and visual impairments [56]. Furthermore, even relatively low-level exposure to MeHg has been demonstrated to be significantly associated with lower regional grey matter volume in the thalamus and hippocampus, and widespread reduction in regional white matter volume, mainly in the frontal lobes bilaterally and right basal ganglia [57].

Particularly, a better understanding about the neurological burden of long-term exposure to MeHg is paramount to indigenous populations living in the Amazon, where levels of exposure have been deemed some of the highest in the world [15,17,58]. These populations have been subject to environmental exposure to MeHg for over 40 years mostly due to illegal gold mining activities in the region [7]. However, data about mean mercury levels in hair samples are only available since 1995, and vary from 2.0 to 75.5 μg/g, according to the time period and studied region [59]. Furthermore, the large majority of studies were conducted within the Tapajos river basin, located in the southern region of the Amazon basin, and encompassing only about 7% of its full extension [15]. Also, while most studies have reported on total mercury levels, only a small number have addressed specifically MeHg [15]. Nonetheless, these studies have described significant associations between high mercury levels and several neurological disorders, mostly compromising motor, color vision, and somatosensory functions [15]. Contrastingly, despite also finding an association between higher MeHg levels (i.e., >6.0 μg/g) and somatosensorial disorders, more specifically altered nociception (*p* = 0.027); our study was not able to detect an association between higher levels of MeHg and the presence of motor deficits. This may be explained by the relatively lower degree of mean mercury exposure observed in our population, which is in the lower range of previously published literature [15,59]; but also by the abovementioned variations of susceptibility to MeHg toxicity, which may also differ at populational levels. On the other hand, our study highlighted associations between MeHg > 6.0 μg/g and peripheral neuropathy, as well as cognitive impairment. Although such associations have been described previously, these neurological outcomes were systematically addressed by very few studies [60,61,62].

While peripheral neuropathy habitually occurs after elemental or inorganic mercury poisoning due to occupational exposure, it has been described in some cases of chronic environmental intoxication with MeHg [63]. Furthermore, it is possible that many cases of MeHg-related peripheral neuropathy may go underdiagnosed, as a previous cross-sectional study in the United States has found that 18% of individuals with idiopathic axonal neuropathy and 9% of those with small fiber neuropathy had blood mercury levels > 10 μg/L [64]. In the selected population of general young and healthy individuals, we found a frequency of 30.3% of peripheral neuropathy, well above the estimated global prevalence of 11.8% [65,66]. Moreover, this condition was significantly associated with a MeHg > 6.0 μg/g. Notably, peripheral neuropathy was also associated with older mean age (Table 2), and each additional year of life predicted a 2.6% (95%CI 1.4–3.7%) increase in its frequency (Table 4). This finding may suggest that age was a confounding factor in the relation between MeHg levels and peripheral neuropathy, as the older the individual, the longer he or she would have been subject to environmental MeHg exposure. Also, older age has been previously established as an independent risk factor for peripheral neuropathy [67,68]. However, there was no correlation between age and hair MeHg levels in our sample, and no evidence of multicollinearity was found between these variables, which makes this possibility less likely. The frequency of MeHg-related peripheral neuropathy has been very rarely addressed in the Amazon indigenous populations. To the best of our knowledge, the only other study to have systematically surveyed this condition, with well-established diagnostic criteria, was conducted in the Sawré Muybu IL, in the Tapajos river basin [62]. It also found a high prevalence (i.e., 43.5%) of peripheral neuropathy, but could not establish an association with the levels of MeHg in hair samples [62].

Meanwhile, the impact of chronic low-level exposure to MeHg on cognition has been reported previously by observational research in various regions of the world [69,70,71,72]. Early-life MeHg poisoning may interfere in neurodevelopment, as both prenatal and postnatal MeHg biomarkers have been revealed to be associated with lower cognitive performance among children [70,71]. Notably, several studies with indigenous Amazonian children also support an association between hair MeHg levels and reduced scores in cognitive and neurodevelopmental scales [16,73,74,75,76]. Furthermore, MeHg has been implied to lead to neurodegeneration, as it may induce hallmark neuropathological changes seen in Alzheimer’s disease [77,78]. In fact, a prospective cohort with 2136 subjects demonstrated that baseline serum mercury levels were associated with the incidence of mild cognitive impairment, after correcting for fish oil intake (OR 2.56; 95%CI 1.003–6.5) [69]. However, the association between MeHg and cognitive disorders among adults in the Amazon IL has only been addressed by few studies, but with positive findings [60,61,62]. One of the largest of these observed significantly higher frequencies of cognitive abnormalities among individuals with hair MeHg concentrations > 10.0 μg/g, as assessed by the BCSB and S-VFT. These data fall largely in line with those found in our sample. Moreover, we observed that a reduced cognitive performance was associated with lower serum levels of Hb (Table 3 and Table 4), as well as of systolic and diastolic blood pressures (Table 3). Further research is needed to better explore the nature of these findings, but they may reflect the consequences of MeHg poisoning in hematological [79] and cardiovascular systems [80]. Notably, however, no evidence of multicollinearity between these factors and MeHg levels, which could support this hypothesis, was found. Interestingly, no association was found between age and cognitive impairment, although the former is a well-established risk factor for dementia and other cognitive disorders [81,82]. This may be explained by the low proportion (7.1%) of elderly individuals in our sample.

It is important to highlight that the findings of this research should be interpreted with caution taking in consideration its limitations. Firstly, this was a cross-sectional study, and therefore the observed associations between hair MeHg levels and neurological outcomes do not establish causality. In fact, bias due to reverse causality and unaddressed confounding factors cannot be excluded. Therefore, further prospective studies are needed to confirm and better explore the nature of these associations. Secondly, our sample was composed mostly by relatively young people, as only 7.1% were ≥65 years old. This may limit the generalizability of our findings for the indigenous elderly population. Thirdly, as only 43% of the total population living in the studied region volunteered to participate in this research, there is a risk for selection bias in our sample. For example, elderly and handicapped people living in more distant villages, as well as those busy engaged in laboring activities (e.g., fishing, agriculture, and hunting) may not have been able to enroll. On the other hand, individuals who suffered from neurological symptoms or who considered themselves more likely to have been exposed to MeHg contamination, would have been more interested to volunteer, given the scope of our work. The relatively small sample size may also have rendered some of the statistical analyses underpowered. Notably, this is a common limitation of research in this context, reflecting the small sizes of the indigenous villages inhabiting the Amazon basin, and the limitations of transportation within this area. Nonetheless, this is one of the largest study published in this field, and possibly the largest to have conducted a systematic detailed neurological evaluation of the studied population, according to a standardized protocol. Furthermore, although the case definition of peripheral neuropathy was made based on well-established criteria [37], no data about complementary neurophysiological testing were available to support the diagnosis. Finally, even though psychiatric symptoms and visual abnormalities have been described in association with MeHg toxicity, they were not systematically surveyed in this study.

In conclusion, the data from this research contribute to better characterize the severity and spread of the consequences of long-term mercury contamination in the Amazon River basin to the indigenous population. When considered together with findings from previous studies in this and other regions of the world, it indicates that long-term environmental MeHg poisoning, even at relatively low concentrations, may contribute significantly to the development of peripheral neuropathy and cognitive impairment, both conditions which may be irreversible and very debilitating. It also contributes to the body of evidence that supports the urgent need for systematic surveillance and regulation of activities in the Amazon region that may lead to the implementation of legislative measures to reduce the continued environmental contamination with mercury.

## Figures and Tables

**Table 1:** Demographic characteristics and neurological examination findings between subjects with MeHg levels > 6.0 μg/g and those with levels ≤ 6.0 μg/g in the Yanomami indigenous population, 2022.

	MeHg ≤ 6.0 μg/g (n = 137)	MeHg > 6.0 μg/g (n = 16)	*p*
Female gender	79 (57.7%)	8 (50.0%)	0.558
Age (years) ^A^	31.55 ± 16.84 (12–75.7)	35.18 ± 18.45 (12.5–69.8)	0.315
Monthly income (R$) ^A^	1345.82 ± 956.04 (0–3450)	1356.88 ± 828.55 (350–3000)	0.956
BMI (kg/m^2^) ^A^	22.62 ± 3.0 (17.1–33.1)	22.4 ± 2.9 (18.8–28.8)	0.672
SBP (mmHg) ^A^	108.68 ± 11.6 (83–155.5)	106.6 ± 10.88 (91–130.0)	0.497
DBP (mmHg) ^A^	70.67 ± 9.92 (41.5–98.5)	68.09 ± 7.76 (54.5–83.0)	0.313
Abnormal blood pressure ^B^	3 (2.2%)	0 (0%)	1.000
Hb (mg/dL) ^A^	13.57 ± 1.38 (10.4–17.2)	13.92 ± 1.22 (11.8–16.1)	0.360
Serum glucose levels (mg/dL) ^A^	94.02 ± 18 (56–150)	96.88 ± 16.2 (71–128)	0.427
Serum glucose > 126 mg/dL	19 (13.9%)	2 (12.5%)	1.000
Previous medical conditions	14 (10.2%)	3 (18.8%)	0.391
Abnormal verbal fluency test	46 (33.6%)	7 (43.8%)	0.418
Verbal fluency test score ^A^	14.8 ± 5.7 (4–30)	13.7 ± 5.9 (7–25)	0.522
Abnormal late recall test	8 (7.1%)	1 (10%)	0.546
Delayed recall score ^A^	8.2 ± 1.2 (3–10)	8.1 ± 1.4 (6–10)	0.678
Abnormal cognitive testing ^C^	46 (35.9%)	8 (66.7%)	0.059
Motor deficit	3 (2.2%)	0 (0%)	1.000
Toe amyotrophy	5 (3.6%)	1 (6.2%)	0.491
Abnormal gait	11 (8%)	1 (6.2%)	1.000
Abnormal tonus	2 (1.5%)	0 (0.0%)	1.000
Bradykinesia	2 (1.5%)	0 (0%)	1.000
Abnormal ankle reflex	22 (16.1%)	4 (25.0%)	0.478
Distal sensory deficit	18 (13.1%)	3 (18.8%)	0.463
Abnormal nociception	19 (13.9%)	6 (37.5%)	0.027 *
Thermal sensory deficit	21 (15.3%)	2 (12.5%)	1.000
Abnormal deep sensory	10 (7.3%)	3 (18.8%)	0.140
Peripheral neuropathy	38 (42.1%)	9 (56.2%)	0.041 *
Speech disturbance	1 (0.7%)	0 (0%)	1.000
Visual field deficits	1 (0.7%)	0 (0%)	1.000

Values presented as n (%), unless stated otherwise. ^A^ Values presented as mean ± standard deviation (minimum–maximum). ^B^ An abnormal blood pressure was considered to be a systolic arterial pressure ≥ 140 mmHg and/or a diastolic blood pressure ≥ 90 mmHg. ^C^ An abnormal cognitive testing was considered to be a score < 6 at the delayed recall phase of the Brief Cognitive Screening Battery or a score < 9 at the verbal fluency test. * *p* < 0.05. SBP—systolic blood pressure; DBP—diastolic blood pressure; MeHg—methylmercury; BMI—body mass index; SBP—systolic blood pressure; DBP—diastolic blood pressure; Hb—hemoglobin levels.

**Table 2:** Demographic characteristics and laboratory findings between subjects with and without peripheral neuropathy of the Yanomami indigenous population, 2022.

	Peripheral Neuropathy Present (n = 47)	Peripheral Neuropathy Absent (n = 107)	*p*
Female gender ^A^	27 (57.4%)	26 (56.1%)	0.874
Age (years)	39.7 ± 20.1 (12–75.7)	27.1 ± 13.6 (12–71.5)	<0.001 *
Monthly income (R$)	1270.39 ± 784.70 (0–2980)	1322.50 ± 1006.28 (0–3450)	0.782
BMI (kg/m^2^)	22.9 ± 3.35 (17.6–33.1)	22.3 ± 2.9 (13.2–31.5)	0.303
SBP (mmHg)	111.4 ± 12.4 (93.5–155.5)	107.1 ± 10.8 (83–139.5)	0.064
DBP (mmHg)	71.81 ± 9.1 (53.5–98.5)	69.7 ± 9.9 (41.5–92.5)	0.293
Abnormal blood pressure ^A,B^	2 (4.3%)	1 (1%)	0.226
Hb (mg/dL)	13.7 ± 1.5 (10.9–17.2)	13.6 ± 1.32 (10.4–16.7)	0.942
Serum glucose levels (mg/dL)	93.8 ± 14.7 (70–130)	94.4 ± 19.0 (56–150)	0.799
Serum glucosis > 126 mg/dL ^A^	5 (10.6%)	17 (15.9%)	0.391
Abnormal cognitive testing ^A,C^	21 (47.7%)	33 (34.4%)	0.132
Hair MeHg levels (μg/g)	3.7 ± 1.4 (1.2–7.0)	4.3 ± 2.1 (1.4–10.1)	0.162
Hair MeHg > 2 μg/g	40 (85.1%)	94 (88.7%)	0.536
Hair MeHg > 6 μg/g	9 (19.1%)	7 (6.6%)	0.041 *

Values presented as mean ± standard deviation (minimum–maximum), unless otherwise specified. ^A^ Values presented as n (%). ^B^ An abnormal blood pressure was considered to be a systolic arterial pressure ≥ 140 mmHg and/or a diastolic blood pressure ≥ 90 mmHg. ^C^ An abnormal cognitive testing was considered to be a score < 7 at the late recall test or a score < 12 at the verbal fluency test. * *p* < 0.05. MeHg—methylmercury; BMI—body mass index; SBP—systolic blood pressure; DBP—diastolic blood pressure; Hb—hemoglobin levels; Hg—mercury.

## Data Availability

The original contributions presented in the study are included in the article, further inquiries can be directed to the corresponding authors.

## Supplementary Materials

The following supporting information can be downloaded at: https://www.mdpi.com/article/10.3390/toxics12030212/s1, Table S1. Demographic characteristics and neurological examination findings between subjects with mercury levels > 2.0 μg/g and those with levels ≤ 2.0 μg/g in the Yanomami indigenous population, 2022.
