# Supplementary material for: Long-Term Environmental Methylmercury Exposure Is Associated with Peripheral Neuropathy and Cognitive Impairment among an Amazon Indigenous Population
Source: Toxics. 2024 Mar 12;12(3):212. doi: 10.3390/toxics12030212 (PMC11154458; doi:10.3390/toxics12030212)
Supplement: Supplementary file 1 [file toxics-12-00212-s001.zip › toxics-2867152-suppplementary.pdf]

Table S1. Demographic characteristics and neurological examination findings between subjects with mercury levels > 2.0 μg/g and those with levels ≤ 2.0 μg/g in the Yanomami indigenous population, 2022.

	**MeHg ≤ 2.0 μg/g (n=19)**	**MeHg > 2.0 μg/g (n=134)**	**p**
Female gender	9 (47.4%)	78 (58.2%)	0.372
Age (years)[A]	22.6 ± 12.6 (12-57.8)	32.2 ± 17.0 (12-75.7)	0.008*
Monthly income (R$)[A]	1366.16 ± 915.87 (400-2889)	1301.61 ± 950.28 (0-3450)	0.733
BMI (mmHg)	22.4 ± 3.35 (17.09-29.81)	22.6 ± 2.95 (17.21-31.13)	0.896
SBP (mmHg)	109.89 ± 7.76 (96.5-126)	108.16 ± 11.88 (83-155.5)	0.540
DBP (mmHg)	73.65 ± 7.42 (59-88)	69.94 ± 9.89 (41.5-98.5)	0.118
Abnormal blood pressure[B]	0 (0%)	3 (2.3%)	1.000
Hb (g/dl)[A]	13.58 ± 1.31 (11.8-16.4)	13.63 ± 1.37 (10.4-17.2)	0.746
Serum glucose (mg/dl)[A]	101.74 ± 18.50 (80-150)	93.07 ± 17.33 (56-145)	0.070
Serum glucose > 126mg/dl	2 (10.5%)	19 (14.2%)	1.000
Abnormal verbal fluency test	5 (26.3%)	48 (35.8%)	0.386
Verbal fluency test score[A]	14.7 ± 6.8 (4-26)	13.8 ± 5.4 (4-30)	0.663
Abnormal late recall test	3 (1.3)	6 (7.7)	0.126
Delayed recall score[A]	8.0 ± 1.2 (6-10)	8.24 ± 1.2 (3-10)	0.425
Abnormal cognitive testing[C]	5 (27.8%)	49 (40.2%)	0.314
Motor deficit	1 (5.3%)	2 (1.5%)	0.330
Toe amyotrophy	0 (0%)	6 (4.5%)	1.000
Abnormal gait	1.5 (0%)	10.5 (9%)	0.364
Abnormal tonus	0 (0%)	2 (1.5%)	1.000
Bradykinesia	0 (0%)	2 (1.5%)	1.000
Abnormal ankle reflex	5 (26.3%)	21 (15.7%)	0.323
Distal sensory deficit	2.6 (15.8%)	18.4 (13.4%)	0.727
Abnormal nociception	3 (15.8%)	22 (16.4%)	1.000
Thermal sensory deficit	3 (15.8%)	20 (14.9%)	1.000
Abnormal deep sensory	0 (0%)	13 (9.7%)	0.372
Peripheral neuropathy	7 (36,8%)	40 (29.9%)	0.598
Speech disturbance	0 (0%)	1 (0.7%)	1.000
Visual Field Deficits	0 (0%)	1 (0.7%)	1.000

Values presented as n (%), unless stated otherwise.

[A] *Values presented as mean ± standard deviation (minimum-maximum)*

[B] *An abnormal blood pressure was considered to be a systolic arterial pressure ≥ 140mmHg and/or a diastolic blood pressure ≥ 90mmHg*

[C] *An abnormal cognitive testing was considered to be a score < 6 at the delayed recall phase of the Brief Cognitive Screening Battery or a score < 9 at the verbal fluency test.*

MeHg – Methylmercury; BMI – body mass index SBP – systolic blood pressure; DBP – diastolic blood pressure; Hb – hemoglobin levels;
